# Clinical and molecular characteristics of myotonia congenita in China: Case series and a literature review

**DOI:** 10.1080/19336950.2022.2041292

**Published:** 2022-02-16

**Authors:** Yifan Li, Mao Li, Zhenfu Wang, Fei Yang, Hongfen Wang, Xiujuan Bai, Bo Sun, Siyu Chen, Xusheng Huang

**Affiliations:** a Geriatric Neurological Department of the Second Medical Center & National Clinical Research Center for Geriatric Diseases, Chinese Pla General Hospital, Beijing, China; b Department of Neurology of the First Medical Center, Chinese Pla General Hospital, Beijing, China

**Keywords:** Myotonia congenita, *CLCN1*, novel mutations, genotype, phenotype

## Abstract

Myotonia congenita (MC) is a rare genetic disease caused by mutations in the skeletal muscle chloride channel gene (*CLCN1*), encoding the voltage-gated chloride channel ClC-1 in skeletal muscle. Our study reported the clinical and molecular characteristics of six patients with MC and systematically review the literature on Chinese people. We retrospectively analyzed demographics, clinical features, family history, creatine kinase (CK), electromyography (EMG), treatment, and genotype data of our patients and reviewed the clinical data and *CLCN1* mutations in literature. The median ages at examination and onset were 26.5 years (range 11–50 years) and 6.5 years (range 1.5–11 years), respectively, in our patients, and 21 years (range 3.5–65 years, n = 45) and 9 years (range 0.5–26 years, n = 50), respectively, in literature. Similar to previous reports, myotonia involved limb, lids, masticatory, and trunk muscles to varying degrees. Warm-up phenomenon (5/6), percussion myotonia (3/5), and grip myotonia (6/6) were common. Menstruation triggered myotonia in females, not observed in Chinese patients before. The proportion of abnormal CK levels (4/5) was higher than data from literature. Electromyography performed in six patients revealed myotonic changes (100%). Five novel *CLCN1* mutations, including a splicing mutation (c.853 + 4A>G), a deletion mutation (c.2010_2014del), and three missense mutations (c.2527C>T, c.1727C>T, c.2017 G > C), were identified. The c.892 G > A (p.A298T) mutation was the most frequent mutation in the Chinese population. Our study expanded the clinical and genetic spectrum of patients with MC in the China. The MC phenotype in Chinese people is not different from that found in the West, while the genotype is different.

## Introduction

Myotonia congenita (MC) is a genetic disease caused by mutations in *CLCN1* on chromosome 7q35 (OMIM # 118,425), coding for the main voltage-dependent chloride channel CLC-1 in skeletal muscle cells [1]. The most characteristic symptom is impaired muscle relaxation after forceful contraction (myotonia). Stiffness worsens after rest and improves with repeated activity (warm-up phenomenon). The most common stiffness sites are the legs, while the face is less commonly affected [2].

The disease can be distinguished into the autosomal dominant-type Thomsen (TMC) (OMIM #160,800) and the autosomal recessive-type Becker (BMC) (OMIM #255,700) by genetic findings and inheritance patterns [3]. There were 348 pathogenic mutations throughout the *CLCN1* gene identified in the Human Gene Mutation Database (HGMD; http://www.hgmd.cf.ac.uk/ac/index.php) to date. TMC is usually caused by a heterozygous mutation on one allele, while compound heterozygous mutations inherited from each parent are common in BMC patients. Some mutations may lead to autosomal dominant MC in some families and a recessive form in others [4,5]. Certain clinical features may help differentiate TMC from BMC. TMC may be associated with milder characteristics and age of onset tends to be earlier in this type [5,6]. However, the phenotype can differ significantly between siblings or children and parents [7]. The genotypic and phenotypic heterogeneity of the disease creates challenges in distinguishing between dominant and recessive types distinctly, especially in sporadic cases or small kindreds. A classic Mendelian pattern does not always elucidate myotonia congenita; terms, such as incomplete dominance with variable penetrance or expressivity, better describe the modes of transmission in some families [8].

With the development of gene sequencing, especially the next-generation sequencing (NGS) technology, increasing cases of genetically confirmed MC have been reported in China since 2010. All studies in the Chinese population have been case reports or case series to date. And a recent literature review found that there was no significant difference in main clinical presentation between the two types of MC, however other aspects of the diseases, such as triggers, CK levels and treatment, were not fully discussed [9]. We analyzed the data on genetically confirmed MC diagnosed in our center in detail, and compared our results with the systematic literature review on the clinical features of all patients with MC, including probands and their affected family members, regardless of inheritance pattern. To describe the mutation spectrum of *CLCN1* in the Chinese population more accurately, the same mutation with different expressions were identified.

## Methods

### Patients

Fourteen patients referred to the Neurology Clinic of the Chinese PLA General Hospital were diagnosed with nondystrophic myotonias (NDMs) by clinical and electrophysiological features from October 11^th^, 2010, to October 21^st^, 2020. Of these, six had a genetically confirmed diagnosis of MC, and one was diagnosed with paramyotonia congenita. We obtained a complete medical history of the patients; an experienced neurologist performed physical and neurological examinations. Each patient with MC received a telephone follow-up on September 2021. The study was approved by the Ethics Committee of the Chinese PLA General Hospital. All patients provided written informed consent.

We performed a detailed search of all relevant English language literature published between 1960 and 2021 via PubMed (http://www.ncbi.nlm.nih.gov/pubmed), using “myotonia congenita” and “Chinese” as keywords. Chinese literature was also searched via CNKI (www.cnki.net), using “myotonia congenita” as keywords. The diagnosis of all patients was supported by molecular genetic testing. Clinical data and mutations were recorded by investigating original articles. Four MC patients comorbid with other genetic diseases (one patient with hypokalemic periodic paralysis type 1, one patient with paroxysmal kinesigenic dyskinesia, one patient with Hoffmann syndrome) were excluded from clinical analysis. We included every pedigree with *CLCN1* mutations to obtain the complete picture of the mutation spectrum. We enrolled 58 MC patients and 47 pedigrees in the literature review (Table 1).

**Table 1.** Mutational spectrum of the CLCN1 gene in the 47 pedigrees reported in Chinese population

Authors and year	N of pts*	Inheritance pattern	Mutation 1	Mutation 2	Mutation 3
Jou SB, et al 2004 [26]	4	AD	c.929C>T (p.T310M)		
		AD	c.1412C>T (p.S471F)		
		AD/AR	c.1444 G > A (p.G482R)		
		AR	c.1723C>T (p.P575S)	c.1931A>G (p.D644G)	
Kuo HC, et al 2006 [10]	4	AD	c.2330delG (p.G777Afs*17		
Burgunder JM, et al 2008 [25]	3	AR	c.1744A>T/ c.1657A>T^#^ (p.I553F)	c.1750C>A/ c.1663C>A^#^ (p.H555N)	
		AD	c.2617C>T (p.L844F)		
Gao F, et al 2010 [11]	2	AD	c.892 G > A (p.A298T)		
		AR	c.782A>G (p.Y261C)	c.1679 T > C (p.M560T)	
Ma FC, et al 2011 [12]	1	AD	c.892 G > A (p.A298T)		
Chen ZT, et al 2012 [27]	2	AD	c.1024 G > A /c.937 G > A^#^ (p.A313T)		
		AD/AR	c.1205 C > T (p.A402V)		
Kong LN, et al 2012 [13]	1		c.950 G > A (p.R317Q)	c.1205C>T (p.A402V)	
Li HF, et al 2014 [14]	0		c.1723C>T (p.P575S)	c.2492A>G (p.Q831R)	
Liu XL, et al 2015 [15]	5	AD	c.782A>G (p.Y261C)	c.2576 G > A (p.G859D)	
		AD	c.1568 G > A (p.G523D)		
		AR	c.1679 T > C (p.M560T)	c.1679 T > C (p.M560T)	
		AR	c.1679 T > C (p.M560T)	c.2364 + 2 T > C (splicing)	
		AR	c.139C>T (p.R47W)	c.685 G > A (p.V229M)	
Meng YX, et al 2016 [41]	8	AR	c.829 T > C (p.C277R)	c.1012C>T (p.R338X)	
		AD	c.1262insC (p.R421Pfs*9X)		
		AR	c.892 G > A (p.A298T)	c.1872 G > T (p.E624X)	
		AR	c.1389insT (p.V465Rfs*44)	c.2330delG (p.G777Afs*17)	
		AR	c.892 G > A (p.A298T)	c.214_215delAG (p.R72Gfs*21)	
		AD	c.2362C>T (p.Q788X)		
Yang XL, et al 2017 [4]	5	AD	c.871 G > A (p.E291K)	c.2172 + 4A>G (splicing)	
		AR	c.1013 G > A (p.R338Q)	c.139C>T (p.R47W)	
		AD	c.892 G > A (p.A298T)		
		AD	c.892 G > A (p.A298T)		
		AD	c.350A>G (p.D117G)		
Gu P, et al 2017 [16]	1	AR	c.1129C>T (p.R377X)	c.1887delC (p.Q630Rfs*17)	
Miao J, et al 2018 [39]	1	AR	c.1401 + 1 G > A (splicing)	c.1657A>T (p.I553F)	
Jin F, et al 2018 [17]	1	AD	c.937 G > A (p.A313T)	c.1205C>T (p.A402V)	
Zhang W, et al 2019 [18]	1	AR	c.280 G > T (p.D94Y)	c.618C>A (p.Y206*)	
Yang HJ, et al 2019 [19]	7	AD	c.892 G > A (p.A298T)		
		AD/AR	c.2169C>A (p.S723R)		
Zhao CY, et al 2020 [20]	3	AD	c.917 T > C (p.F306S)		
Cao XL, et al 2020 [21]	1	AD	c.1879A>C (p.T627P)		
Su MX, et al 2020 [22]	0	AR	c.2492A>G (p.Q831R)	c.2492A>G (p.Q831R)	
Song J, et al 2021 [23]	3	AD	c.907 T > C (p.W303R)		
		AD	c.762C>G (p.C254W)		
		AR	c.1876C>T (p.R626*)	c.1408A>G (p.M470V)	
Hu C, et al 2021 [9]	5	AR	c.139C>T (p.R47W)	c.1657A>T (p.I553F)	c.892 G > A (p.A298T)
		AD	c.1649C>T (p.T550M)		
		AR	c.962 T > A (p.V321E)	c.350A>G (p.D117G)	
		AR	c.1250A>T (p.E417V)	c.1277C>A (p.T426N)	
		AR	c.762C>G (p.C254W)	c.892 G > A (p.A298T)	

**N of pts, number of patients; AD, autosomal dominant; AR, autosomal recessive**. * The number of patients for clinical analysis. ^#^ The renamed nucleotide position by the commonly used transcript.

### Clinical data collection

The following data were collected and analyzed: demographics, age of onset, distribution of myotonia, other concomitant symptoms (weakness, muscle hypertrophy, myalgia, cramps), myotonia characteristics (warm-up, grip myotonia, percussion myotonia), triggers (cold, stress, exercise, menstruation), elevated CK, family history, anti-myotonic medications, and efficacy.

### Exon sequencing for ion channelopathy genes

For screening potential pathogenic genes for MC, we performed gene panel sequencing for ion channelopathy containing 12 genes (*CACNA1A, CACNA1S, CAV3, CLCN1, KCNA1, KCNE3, KCNE4, KCNH2, KCNJ16, KCNJ2, KCNQ1, SCN4A*) (GenCap Enrichment technologies, MyGenostics Inc, Beijing, China), using HiSeq 2000 high-throughput sequencing system (Illumina Inc, USA). The *DMPK* gene was analyzed in the patient with myopathic changes on EMG.

### Bioinformatics analysis

After filtering out the low-quality reads and adaptor sequences using the Solexa QA package and the Cutadapt program (http://code.google.com/p/cutadapt/), respectively, we used the SOAPaligner program to align the clean read sequences to the human reference genome (hg19). After removing the PCR duplicates by the Picard software, we first identified the SNPs using the SOAPsnp program (http://soap.genomics.org.cn/soapsnp.html). Subsequently, we realigned the reads to the reference genome using BWA and identified the insertions or deletions (InDels) using the GATK program (http://www.broadinstitute.org/gsa/wiki/index.php/Home_Page). The identified SNPs and InDels were annotated using the Exome-assistant program (http://122.228.158.106/exomeassistant). MagicViewer was used to view the short read alignment and validate the candidate SNPs and InDels. Nonsynonymous variants were evaluated by four algorithms, PolyPhen2, SIFT, Mutation Taster, and REVEL (as described previously), to determine pathogenicity. Mutations were searched in the 1000 Genomes Project database (www.1000genomes.org), Exome Aggregation Consortium (ExAC) database (www.exac.broadinstitute.org), Genome Aggregation Database (gnomAD), and dbSNP database (http://www.ncbi.nlm.nih.gov/projects/SNP) followed by the Human Genome Mutation Database (http://www.biobase-international.com/product/hgmd HGMD) and the ClinVar database (https://www.ncbi.nlm.nih.gov/clinvar/) to determine whether the variant was a reported pathogenic mutation.

Sanger sequencing validation

PCR amplification was optimized, following the standard PCR protocol using FastStart Taq DNA Polymerase, dNTPack (Roche Applied Science). A sequencing reaction was performed using the BigDye® v.1.1 Terminator cycle sequencing kit and the ABI Prism® 3130xl Genetic Analyzer (Life Technologies).

### Statistical analysis

Statistical analysis was performed with IBM SPSS Statistics 26.0. Descriptive statistics of demographic characteristics were made using medians and range for continuous variables and frequency analysis for categorical variables.

## Results

### Clinical features

The clinical diagnosis of MC was established in all six patients according to the Diagnostic Criteria for Neuromuscular Disorders [24]. The demographics and clinical features are summarized in Table 2.

**Table 2.** Demographics and clinical features of this cohort of patients

Patient	P1	P2	P3	P4	P5	P6
Gender	Female	Female	Male	Male	Male	Male
Age at examination(yr)	34	11	33	20	50	14
Age at onset(yr)	1.5	8	11	5	8	5
Family history	–	–	+	–	–	–
Inheritance pattern based on the pedigree	Sporadic	Sporadic	AR	Sporadic	Sporadic	Sporadic
Symptoms at onset	LL stiffness	LL stiffness	LL stiffness	LL+UL stiffness	LL stiffness	LL+UL stiffness
Distribution of myotonia						
Lid	–	–	+	NA	+	–
Masticatory muscles	+	–	+	NA	NA	+
Trunk	+	–	–	–	–	–
Upper limb	+	+	+	+	+	+
Lower limb	+	+	+	+	+	+
Other concomitant symptoms						
Weakness	–	–	–	–	–	–
Muscle hypertrophy	–	+	–	–	–	+
Myalgia	–	–	–	–	–	+
Cramps	–	+	–	NA	–	–
Dysphagia	–	–	+	NA	–	–
Dysphonia	–	–	–	NA	–	–
Myotonia characteristics						
Warm-up	+	+	+	+	+	–
Grip myotonia	+	+	+	+	+	+
Percussion myotonia	+	+	–	NA	+	–
Triggers						
Cold	+	+	+	+	+	+
Stress	+	+	+	NA	–	–
Exercise	–	–	–	NA	–	–
Alcohol	–	–	–	NA	–	–
Menses	+	+	NA	NA	NA	NA
CK (U/L)	NA	79.7	309.7	275.2	380.1	579.6
Medication	phenytoin	phenytoin	–	–	–	–
Response to treatment	–	+	NA	NA	NA	NA
Electromyography						
Myotonic discharges	+++	+++	+++~++++	+~++	+	+++
Myopathic changes	–	–	+	–	–	–
Prognosis	stable	stable	stable	NA	relieved	relieved

yr, years; LL, lower limbs; UL, upper limbs; NA, not applicable.

**Figure 1.**
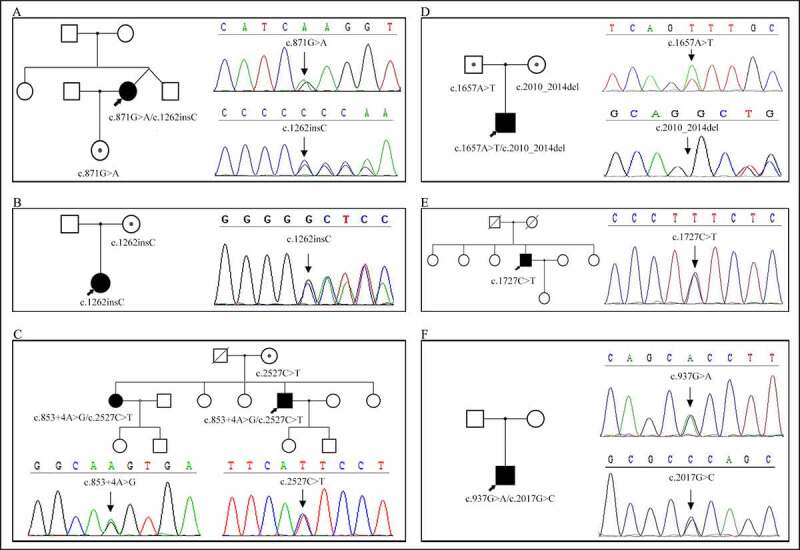
The pedigree and genetic analysis of the six patients with myotonia congenita. a. Patient 1, *CLCN1* c.871 G > A (p.E291K) and c.1262insC (p.R421PfsX9) mutation. b. Patient 2, *CLCN1* c.1262insC (p.R421PfsX9) mutation. c. Patient 3, *CLCN1* c.853 + 4A>G (splicing) and c.2527C>T (p.L843F) mutation. d. Patient 4, *CLCN1* c.1657A>T (p.I553F) and c.2010_2014del (p.L671Rfs*41) mutation. e. Patient 5, *CLCN1* c.1727C>T (p.S576F) mutation. f. Patient 6, *CLCN1* c.937 G > A (p.A313T) and c.2017 G > C (p.A673P) mutation.

There were four males and two females, with median ages 26.5 years (range 11–50 years) at examination and 6.5 years (range 1.5–11 years) at the onset. No family history was reported, except in patient 3. We observed similar symptoms in one of his elder sisters but not in other family members (Figure 1c). Stiffness in the legs was more prominent in four patients at the early stage, involving all four limbs eventually. Lids, masticatory muscles, and trunk muscles were also affected (2/5, 3/4, 1/6, respectively). No patient experienced muscle weakness. Patient 2 had slight muscle hypertrophy in the buttocks and thigh; patient 6, a fitness coach, had a muscular appearance. Only one patient reported myalgia, muscle cramps, and dysphagia. No patient reported dysphonia. Myotonia improved with exercise (warm-up phenomenon) in five of the six patients. Grip myotonia was detectable in all patients and percussion myotonia in half of the patients. Myotonia was most commonly exacerbated by cold and stress and became apparent during menstruation in females. No patient had exercise-induced myotonia. The CK levels were 1.4–2.9 folds (range 275.2–579.6 U/L) higher than the upper limit of the normal reference level in four patients. We prescribed phenytoin to four patients, but only two patients took the medication; one patient showed a response to the treatment. Two patients (patients 5 and 6) had mild symptoms and required no pharmacological intervention. Patient 3 could minimize his symptoms by avoiding situations and stimuli that triggered myotonic episodes. Needle EMG revealed myotonic discharges in all patients, with additional myopathic changes in patient 3, whose muscle biopsy (left biceps brachii) showed scattered muscle fiber atrophy. No expansion of CTG repeats was found in the *DMPK* gene. At follow-up, the symptoms were stable in patients 1 to 3 and relieved without treatment in patients 5–6. Patient 1 discontinued the medication due to low efficacy. Patient 2 used the medication intermittently on aggravation of symptoms during winter.

### CLCN1 mutations

Gene panel testing showed that the six patients carried 10 mutations in the *CLCN1* gene; of these, five mutations were novel and not reported elsewhere (Table 3). According to the mutated genes, three cases with compound heterozygous mutations were in the AR inheritance pattern. The inheritance patterns in other cases were uncertain. Patient 1 harbored two compound heterozygous mutations, c.871 G > A (p.E291K) and c.1262insC (p.R421PfsX9). The mutation c.1262insC (p.R421PfsX9) was also detected in patient 2, but the heterozygous state. Patient 3 had two novel heterozygous mutations in the *CLCN1* gene, a splicing mutation (c.853 + 4A>G) and a missense mutation (c.2527C>T), leading to abnormal splicing and an L843F amino acid change, respectively. Molecular analysis of patient 4 revealed a novel compound heterozygous c.2010_2014del (p.L671Rfs*41) mutation in exon 17 and a previously noted c.1657A>T (p.I553F) mutation in exon 14 [25]. The new mutation was a 5 base pair deletion of nucleotides, 2010 to 2014, leading to a frame-shifted sequence beginning with codon 670 and ending with a stop at codon 711. We detected a novel heterozygous transition in nucleotide position 1727 (codon 198) of C to T, resulting in an amino acid change of serine to phenylalanine in patient 5. We identified two heterozygous variants, c.937 G > A (p.A313T) in exon 8 and c.2017 G > C (p.A673P) in exon 17 in patient 6.

**Table 3.** Mutations associated with nondystrophic myotonia identified in the study

patient	Gene	Exon	Nucleotide change	Amino acid change	Mutation type	State	Inheritance pattern based on the mutated gene
1	CLCN1	8 12	c.871 G > A c.1262insC	p.E291K p.R421Pfs*9	Missense Frameshift	Compound heterozygous	AR
2	CLCN1	12	c.1262insC	p.R421Pfs*9	Frameshift	Heterozygous	AD/AR
3	CLCN1	Intronic 22	c.853 + 4A>G * c.2527C>T *	Splicing change p.L843F	Splicing Missense	Compound heterozygous	AR
4	CLCN1	15 17	c.1657A>T c.2010_2014del *	p.I553F p.L671Rfs*41	Missense Frameshift	Compound heterozygous	AR
5	CLCN1	15	c.1727C>T *	p.S576F	Missense	Heterozygous	AD/AR
6	CLCN1	8 17	c.937 G > A c.2017 G > C*	p.A313T p.A673P	Missense Missense	Compound heterozygous	AD/AR

* novel mutation

### Pathogenicity analysis

The pathogenicity of the five novel mutations was predicted using Mutation Taster, PolyPhen-2, SIFT, and REVEL. The results are listed in Table 4. According to the bioinformatics analysis results and the American College of Medical Genetics and Genomics (ACMG) guidance for the interpretation of sequence variants, we considered these five mutations as potential pathogenic variants.

**Table 4.** Bioinformatics analysis of the novel mutations of CLCN1 gene

	Mutation Taster	Polyphen-2	SIFT	REVEL	Variant Classification (ACMG)
c.853 + 4A>G	-	-	-	-	Uncertain
c.2527C>T	Disease_causing	Probably_damaging	Damaging	D	Likely pathogenic
c.2010_2014del	-	-	-	-	pathogenic
c.1727C>T	Disease_causing	Probably_damaging	Damaging	D	Uncertain
c.2017 G > C	Polymorphism	Benign	Tolerated	B	Uncertain

### Pedigree study

Sanger sequencing was performed in the probands and family members of four patients (patients 1, 2, 3, 4) to determine whether their relatives carried the same mutation as the proband. The daughter of patient 1, whose neurological examination and EMG findings were normal, carried a heterozygous mutation c.871 G > A, but not the c.1262insC (p.R421PfsX9) mutation (Figure 1a). The same mutation (c.1262insC) was also detected in the mother of patient 2 (Figure 1b). However, this truncating mutation showed no clinical manifestation in his mother. The healthy mother of patient 3 had the same missense mutation (c.2527C>T) but a normal sequence at the other position. His affected sister had the same heterozygous mutations (c.853 + 4A>G, c.2527C>T) as the proband (Figure 1c). Both parents of patient 4 were heterozygous carriers of the mutations and remained unaffected clinically. His mother carried the c.2010_2014del mutation and his father c.1657A>T the mutation (Figure 1d). Unfortunately, other family members of patients 5 and 6 were unavailable for genetic testing (Figure 1e,f).

### Clinical Findings of the literature

**Figure 2.**
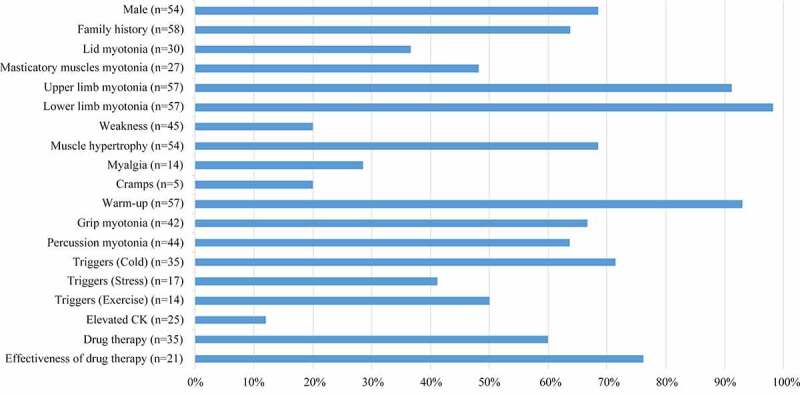
Clinical features of the 58 Chinese patients in literature. (The number in the brackets were the number of patients available for analysis).

In literature, the median ages at examination and onset were 21 years (range 3.5–65 years, n = 45) and 9 years (range 0.5–26 years, n = 50), respectively. According to the clinical phenotype and genotype, 21 pedigrees (52.5%) were TMC, and 16 (40%) were BMC. It was difficult to determine the inheritance pattern of the three sporadic cases with only one heterozygous mutation in *CLCN1*. As shown in Figures 2, 68.5% (37/54) of the patients were male, and 63.8% (37/58) had a family history. The most frequent symptoms were myotonia of the upper and lower limbs (92.3% vs. 98.1%), followed by the warm-up phenomenon. Myotonia at onset most frequently involved lower limbs (76.5%). Other symptoms, including muscle hypertrophy, grip myotonia, and percussion myotonia, were observed in over half of the patients. Cold temperature environment was the most common trigger for myotonia in the reported patients (80%). Other uncommon triggers involved stress and exercise. Elevated CK was reported only in 10% (2/20) of Chinese patients. EMG detected myotonic runs in 96.3% (52/54) of MC patients; myopathic discharges occurred in 13% (7/54) of these patients. Anti-myotonic drugs were effective in 76.2% (16/21) of patients. Patients on mexiletine or carbamazepine (15/30) reported relief of symptoms. However, patients treated with phenytoin (5/30) showed poor response to medication. Only one patient received propafenone, which was effective.

### Mutation spectrum of the Chinese population

**Figure 3.**
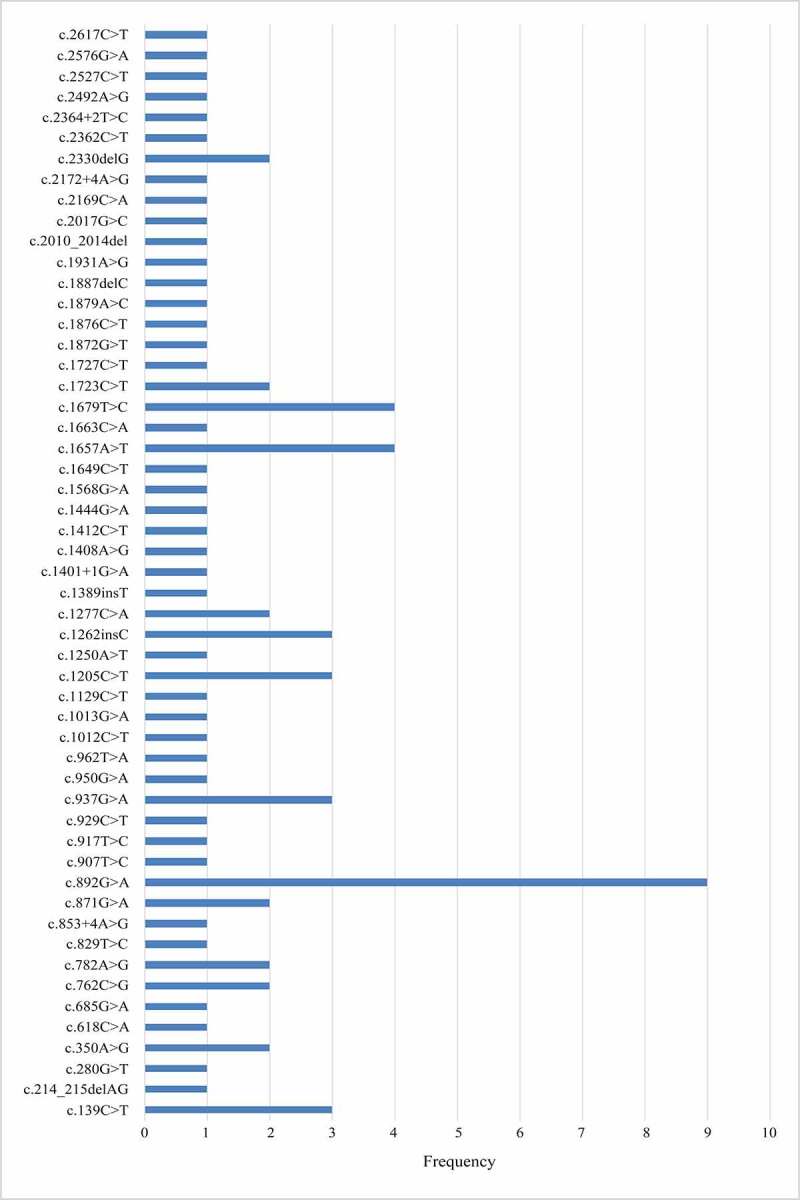
Mutational spectrum of the 53 Chinese pedigrees including our study.

To date, there are 53 Chinese pedigrees reported, including our study. Fifty-three different variants have been found scattered across the 23 exons of the *CLCN1* gene. We identified a region of high mutation frequency that includes exons 8 and 15 of the *CLCN1* gene: 34 (41.5%) unrelated probands harbored mutations in this region; other exons with a high rate of mutations, although to a less frequency, included exons 11, 12, and 17. Of the known mutations, three missense mutations were more frequent: c.892 G > A (p.A298T) (n = 9) in exon 8, 1657A>T (p.I553F) (n = 4), and c.1679 T > C (p.M560T) (n = 4) in exon 15 (Figure 3).

## Discussion

Our study illustrated the clinical characteristics of genetically diagnosed Chinese patients with MC from muscular features, inheritance patterns, and auxiliary examination to treatment. Besides, we identified five novel mutations: c.853 + 4A>G (splicing change), c.2010_2014del (p.L671Rfs*41), c.2527C>T (p.L843F), c.2017 G > C (p.A673P) in the compound heterozygous state, and c.1727C>T (p.S576F) in the heterozygous state, which expanded the genetic spectrum of patients with MC in the Chinese population.

Myotonia congenita is a rare inherited disease. Chinese patients with MC with proven mutations in the *CLCN1* gene were first reported in 2004 by Taiwan scholars [26]. The diagnosis of NDMs was solely based on clinical and electrophysiological findings until next-generation sequencing was used in the clinical setting in our center in 2014. To our knowledge, there was no large cohort of Chinese MC patients have been described. We reprocessed and analyzed the clinical data on the previously reported 58 MC cases as a cohort, and compared it with our clinical findings in family history, muscular features, myotonia triggers, CK levels, and drug therapy. To depict the mutation spectrum of *CLCN1* in the Chinese population, we analyzed pedigrees instead of patients. We checked every mutation carefully to identify the same mutation with different expressions because different transcripts were used. Mutations c.1657A>T (p.I553F), c.1663C>A(p.H555N) and c.937 G > A (p.A313T) were also expressed as c.1744A>T, c.1750C>A [25], and c.1024 G > A [27], respectively. In this case, we renamed the nucleotide position by the commonly used transcript.

Clinical manifestations in our patients were similar to previous reports. Males were slightly more affected than females. We found that myotonia worsened during menstruation in female patients, implying that sex hormones could alter the function of CLC-1 [28]. Menstruation is not reported as a factor worsening symptoms in Chinese people. CK levels increased in about 10% of Chinese patients, while most of our patients (4/5) had abnormal CK. Our data indicated that the elevation of CK levels was common in MC, as reported in a large German cohort [29]. EMG detected myotonic discharges in almost all patients with MC in the literature and all patients in our study, implying that EMG was the most valuable diagnostic tool for identifying patients with MC and indicating the need for genetic testing. Only two patients had no myotonia discharges on EMG at the examination. One patient with normal EMG at 44 years experienced myotonia at the age of 18. The symptoms showed almost complete resolution by 40 years without treatment [25]. The other patient who carried S471F mutations only showed a positive wave and increased recruitment in the EMG [26]. Myopathic motor unit action potentials (MUAPs) occurred in about 13% of patients in previous reports and 17% (1/6) of patients in our study. Noteworthy that all patients with myopathy changes were BMC, which was in agreement with some published reviews of other populations [30]. However, a more systematic quantitative analysis of MUAPs in genetically proven MC found that myopathic MUAPs occurred in more than 20% of muscles in the entire MC group. The incidence of myopathic MUAPs was 21.1% in the BMC subgroup and 30.4% in the TMC subgroup, and they occurred most often in distal limb muscles in BMC and upper limb muscles in the Thomsen MC group [31]. Our patient exhibited low amplitude and short duration MUAPs with interference patterns in proximal and distal muscles of four limbs. The incidence of myopathic MUAPs in Chinese patients with MC, especially in TMC subgroups, was unknown.

From our literature review, 60% of Chinese patients received anti-myotonic drugs. All patients treated with anti-myotonic drugs received monotherapy. Phenytoin and gabapentin were the most effective therapies in recessive *CLCN1*-myotonia [32]. However, recent studies showed that mexiletine and lamotrigine were more effective [33–35], consistent with our findings. However, randomized controlled trials in the Chinese population are challenging due to the rarity and genetic heterogeneity of the disorder. A large prospective cohort study in Chinese patients is needed to obtain accurate data.

According to the clinical phenotype and genotype, MC pedigree with dominant inheritance accounts for about 50% in the Chinese literature, which is higher than the percentage of Western countries (UK 36%, South-Italian 36.8%, French-Canadian 25%) [32,36,37] but lower than another report from Japan (67%) [38]. A comprehensive prevalence study is needed to determine the prevalence of dominant versus recessive in Asia. In our study, patients 1, 3, and 4 who carried compound heterozygous mutations were BMC. Of the six mutations identified in three BMC patients, c.853 + 4A>G, c.2010_2014del, and c.2527C>T mutations were first reported. The mutation c.1657A>T (p.I553F) is reported in the literature as inherited in a recessive mode [25,39]. One previously reported mutation, c.871 G > A (p.E291K), was recessive in one family [40] but dominant in the other [4]. The c.1262insC (p.R421PfsX9) variant is also reported in the literature as inherited in a recessive [40] or dominant mode [41]. Family history was negative for patients 2 and 5 who carried only one mutated allele; therefore, it was difficult to determine their inheritance pattern. The heterozygous c.1262insC mutation presented in patient 2 and her apparently normal mother who haven’t received EMG test, indicating that this mutation could be recessive or dominant with incomplete penetrance, as suggested previously for G230E [40,42], A313T [8], R317Q [40,42], R338Q [43], and R894X [40,43,44]. Patient 5 had a novel heterozygous mutation S576F, but his parents and children showed no myotonia. One cannot exclude the presence of a second (yet undetected) mutation, and therefore, firm conclusions regarding the inheritance of mutations require functional experiments and the study of other family members carrying the same mutation. Patient symptoms showed complete resolution without treatment, which supported the assumption that this mutation had a dominant-negative effect. This assumption needs confirmation by accumulating more pedigrees and further pathophysiological study. Patient 6 carried two compound heterozygous mutations. As mentioned above, mutation A313T is reported as autosomal recessive or dominant with incomplete penetrance. The other mutation, A673P, is reported in the ExAc database (frequency 0.0016) and the dbSNP database as rs200385034. However, there are no reports on its pathogenicity. PolyPhen-2 software predicted the effects of the A673P mutation as “benign,” SIFT software predicted its effects as “tolerated,” and Mutation Taster indicated “polymorphism.” According to the ACMG standards, this variant could be classified as a variant of uncertain significance. The A673P may be a single nucleotide polymorphism not associated with MC, and therefore, the A313T mutation may be an autosomal-dominant mutation in this patient. Alternatively, the A673P mutation had a recessive effect, and the A313T mutation acted as an autosomal-recessive mutation. The patient was the only affected family member and expressed a mild phenotype, which gradually disappeared with age; his healthy parents refused to undergo genetic evaluation.

Of the known mutations, the most frequent mutation is c.892 G > A (p.A298T) in exon 8 (11%), followed by the c.1657A>T (p.I553F) (4.9%) and c.1679 T > C (p.M560T) (4.9%) in exon 15. A298T was associated with both autosomal dominant and recessive inheritance. This mutation was not frequently reported in other European [45–47] or American [48] cohorts. However, it was most common in Japanese cohorts [38]. Further haplotype studies on these families are necessary to determine whether the founder effect plays a role in the higher frequency of the c.892 G > A mutation.

In addition to the five novel mutations of *CLCN1* identified, our study elaborated the clinical manifestations, drug therapy, and molecular characteristics of Chinese patients with MC. However, no systematic research has been conducted in China. Due to the rarity of the disease, multi-center collaboration is an effective strategy to conduct large-scale cohort studies on phenotype–genotype relationships and randomized controlled trials on treatment.

## Data Availability

The data that support the findings of this study are openly available in Dryad at https://doi.org/10.5061/dryad.0cfxpnw42.
